# Local Control After Single-Isocenter Dynamic Conformal Arc SRS for Brain Metastases

**DOI:** 10.3390/cancers17223711

**Published:** 2025-11-20

**Authors:** Maciej Blok, Izabela Zarebska, Izabela Miechowicz, Tomasz Wisniewski, Szymon Ziolkowski, Maciej Harat

**Affiliations:** 1 Franciszek Lukaszczyk Memorial Oncology Center, Department of Neurooncology and Radiosurgery, 85-796 Bydgoszcz, Poland; 2 Bydgoszcz University of Science and Technology in Bydgoszcz, Faculty of Medicine, 85-029 Bydgoszcz, Poland; 3 Poznań University of Medical Sciences, Department of Computer Science and Statistics, 61-701 Poznań, Poland; 4 Ludwik Rydygier Collegium Medicum in Bydgoszcz, Nicolaus Copernicus Universtiy, Department of Oncology and Brachytherapy, 87-100 Toruń, Poland; 5 Ludwik Rydygier Collegium Medicum in Bydgoszcz, Nicolaus Copernicus Universtiy, Department of Oncology, 87-100 Toruń, Poland

**Keywords:** DCA, brain, metastases, SRS, radiosurgery

## Abstract

Purpose: Brain metastases occur in up to 30% of patients with advanced cancer and remain a major clinical challenge. While WBRT was historically the standard treatment, it provided limited survival benefit and significant neurocognitive toxicity, leading to increasing use of stereotactic radiosurgery (SRS). Recent advances with single-isocenter, multi-target (SIMT) dynamic conformal arc (DCA) techniques on modern linear accelerators have enabled efficient treatment of numerous lesions within a single session, though concerns regarding geometric accuracy persist. This study aimed to evaluate local control outcomes and influencing factors following DCA-SIMT radiosurgery in patients with multiple brain metastases. Methods: We retrospectively analyzed 195 brain metastases treated using single-isocenter, multi-target dynamic conformal arc (DCA-SIMT) stereotactic radiosurgery on a Varian TrueBeam LINAC between August 2018 and September 2020. Treatment planning was performed with Brainlab Elements MultiMets software, version 2.0 and image guidance with ExacTrac. Local control was assessed on MRI according to BM-RANO criteria, while radiation-induced contrast enhancements (RICE) were identified using multiparametric MRI. The median follow-up duration was 12 months. Statistical analyses included chi-square and ROC analyses, with *p* < 0.05 considered statistically significant. Results: A total of 195 brain metastases in 37 patients were analyzed. Local control at 6 months was achieved in 93% of lesions, with complete or partial response in 82%. Distance-to-isocenter (DTI), gradient index (GI), and target volume (GTV/PTV) were not associated with local control. In contrast, conformity index (CI) < 1.42 predicted better treatment response (AUC = 0.698, *p* = 0.0006). Margin expansion ≥ 0.5 mm was associated with improved local control (*p* = 0.049), while higher margins did not further improve outcomes. Prescription dose showed no significant impact. The addition of immunotherapy or targeted therapy within 4 months post-SRS significantly increased the likelihood of radiographic response (OR = 2.55, *p* = 0.030), with the strongest association observed in lung adenocarcinoma patients (*p* < 0.001). Conclusions: DCA-SIMT stereotactic radiosurgery achieves high local control in patients with multiple brain metastases. Conformity index, minimal margin expansion, and systemic therapy influenced outcomes, whereas distance-to-isocenter and dose did not. Further validation is needed to optimize margins in high-DTI scenarios.

## Simple Summary

Brain metastases affect up to one in three patients with advanced cancer and remain a major clinical challenge. Whole-brain radiotherapy, once the standard treatment, offers limited survival benefit and often causes cognitive decline. Stereotactic radiosurgery (SRS) has emerged as a more precise option, enabling treatment of multiple tumors while sparing healthy brain tissue. In this study, we evaluated outcomes of a modern single-isocenter, multi-target technique using dynamic conformal arcs (DCA-SIMT) in patients with multiple brain metastases. We found that this approach provided excellent local control of tumors, and that treatment success was influenced by factors such as plan conformity, small margin expansion, and the addition of systemic therapies like immunotherapy. These results suggest that combining advanced radiosurgery with modern systemic treatments may improve disease control for patients with multiple brain metastases.

## 1. Introduction

Brain metastases occur in approximately 20–30% of patients with advanced malignancies, representing one of the most common neurological complications in oncology [1,2,3]. Despite major advances in diagnostics and systemic therapies, both the incidence and mortality associated with cancer continue to rise, with global projections indicating a further substantial increase in the coming decades [4].

Surgical resection remains an important treatment option for carefully selected patients, particularly those with a solitary lesion, significant mass effect, or elevated intracranial pressure unresponsive to corticosteroids. In contrast, patients with multiple brain metastases rarely benefit from surgery, and their management often relies on radiotherapy or systemic treatments [5]. Historically, whole-brain radiotherapy (WBRT) has been the standard of care for such patients, providing modest survival benefit but frequently resulting in neurocognitive decline—especially in memory and executive functions—as demonstrated in randomized trials [6,7,8,9]. Consequently, there has been a growing shift toward focal, less toxic approaches.

Stereotactic radiosurgery (SRS) has become a cornerstone of modern neuro-oncology, offering excellent local control with limited toxicity in appropriately selected patients [10,11]. However, treating multiple lesions remains technically challenging due to prolonged treatment times and increasing planning complexity, particularly in patients with limited neurological reserve.

Recent technological progress has enabled single-isocenter, multi-target (SIMT) radiosurgery using dynamic conformal arcs (DCA) on modern linear accelerators such as TrueBeam. This approach allows for simultaneous irradiation of multiple lesions in a single session, often completing treatment of ten or more metastases within thirty minutes while maintaining submillimeter accuracy through image-guided stereotactic immobilization [12,13]. Nevertheless, concerns persist regarding potential geometric uncertainties for lesions located farther from the isocenter, which may affect dose conformity and local control [14].

In parallel with these technical innovations, the therapeutic landscape for brain metastases has expanded substantially. The introduction of molecularly targeted therapies and immune checkpoint inhibitors has markedly improved intracranial control in selected tumor subtypes such as non-small-cell lung cancer, breast cancer, and melanoma [15,16]. The synergistic potential between systemic therapy and focal irradiation has become an area of growing clinical and biological interest, as combined strategies may enhance both local and distant tumor response.

Moreover, several novel locoregional modalities are under active investigation. The phase III METIS trial (Mehta et al.) evaluated tumor treating fields (TTF) in patients with brain metastases from non-small-cell lung cancer, demonstrating feasibility and a potential delay in intracranial progression following SRS [17]. Similarly, laser interstitial thermal therapy (LITT) has shown promising results for achieving local control in deep-seated or recurrent metastases not amenable to resection or re-irradiation [18]. Furthermore, amplitude-modulated radiofrequency electromagnetic fields (AM RF EMF) represent a novel, noninvasive approach currently being explored for potential antiproliferative and signaling-modulating effects in cancer cells [19].

These advances reflect a broader paradigm shift toward multimodal and biologically individualized management of brain metastases, where precision radiosurgery serves as both a definitive and complementary component of care. Against this background, the present retrospective study was designed to evaluate local control outcomes following single-isocenter dynamic conformal arc (DCA-SIMT) radiosurgery in patients with multiple brain metastases. In addition, we investigated the potential synergistic effect of concurrent or sequential immunotherapy and targeted therapy and identified treatment-planning parameters—such as conformity index (CI) thresholds and margin expansion—that may optimize plan quality, efficiency, and clinical effectiveness in contemporary SRS practice.

## 2. Materials and Methods

This study retrospectively evaluated patients with a total of 195 metastatic CNS lesions were treated at the Department of Neuro-Oncology and Radiosurgery, Franciszek Lukaszczyk Oncology Center in Bydgoszcz, Poland. All participants provided written informed consent for data collection prior to treatment, in accordance with the Central Nervous System Cancer Registry at the Oncology Center in Bydgoszcz. The study was approved by the Bioethics Committee of the Nicolaus Copernicus University in Toruń, Collegium Medicum in Bydgoszcz, Poland (KB720/2018, Approval Date: 27 November 2018). All procedures were performed in compliance with relevant ethical guidelines and regulations.

Patients were eligible for inclusion if they received stereotactic radiosurgery (SRS) using the Dynamic Conformal Arc—Single-Isocenter (DCA-SIMT) technique between August 2018 and September 2020. All consecutive patients treated within this time period who had follow-up MRI available for evaluation were included in the analysis. Patients for whom post-treatment MRI was not available were not considered for inclusion. Exclusion criteria included prior whole-brain radiotherapy (WBRT) or SRS to the same anatomical region, as well as cases involving postoperative irradiation of resected lesions. All patients had a baseline Karnofsky Performance Status (KPS) of at least 70.

Treatments were delivered using a Varian TrueBeam linear accelerator (LINAC), with patient positioning monitored by the ExacTrac system (Brainlab AG, Munich, Germany). Treatment planning was conducted using Brainlab Elements MultiMets SRS software [20,21].

Assessment of local control was performed based on contrast-enhanced MRI scans obtained 6 months after treatment, using a 1.5 Tesla MRI scanner (Siemens Healthineers, Erlangen, Germany). Lesion response was evaluated according to the Brain Metastases Response Assessment in Neuro-Oncology (BM-RANO) criteria by multidisciplinary tumor board. Local control was defined as a complete response, partial response, or stable disease. Radiation-Induced Contrast Enhancements (RICE) was diagnosed based on multiparametric magnetic resonance imaging, including diffusion-weighted imaging, perfusion sequences, and MR spectroscopy, as well as lesion dynamics consistent with the typical course of post-radiation necrosis.

Statistical analysis was performed using Statistica (version 13, TIBCO Software Inc., Palo Alto, CA, USA) and PQStat software (version 1.8.4, PQStat Software, Poznań, Poland). The Shapiro–Wilk test was used to assess normality of data distribution. For variables meeting assumptions of normality and equal variances, comparisons were made using the independent samples *t*-test. If assumptions were not met, the Mann–Whitney U test was used. Associations between categorical variables were examined using the chi-squared test, Fisher’s exact test, or the Fisher–Freeman–Halton test with the Benjamini–Hochberg correction for multiple comparisons. The odds ratio (OR) with 95% confidence intervals was calculated. A *p*-value < 0.05 was considered statistically significant.

## 3. Results

The patient characteristics are detailed in Table 1. Local control was achieved in 93% of lesions treated with SRS. In 82% of cases, the response was classified as either complete or partial. A detailed description of treatment responses is presented in Table 2. The median overall survival was 13.1 months, and the median follow-up duration was 12 months.

### 3.1. Clinical Factors

In the analysis of clinical factors influencing local control of metastatic lesions, no statistically significant association was found between age and lesion control at 6 months post-treatment (*p* = 0.640). Similarly, sex was not significantly associated with local control (Pearson’s chi-squared test, *p* = 0.878). However, histopathological subtype showed a significant relationship with treatment response (Fisher–Freeman–Halton test, *p* = 0.024), with better local control observed in patients with non-small-cell lung cancer.

### 3.2. GTV and PTV

The potential impact of GTV and PTV (expressed in cm^3^) on local control at 6 months post-treatment was analyzed. No statistically significant association was found for either PTV volume (*p* = 0.616) or GTV volume (*p* = 0.612), as assessed by the Mann–Whitney U test.

### 3.3. Conformity Index (CI)

**Figure 1:**
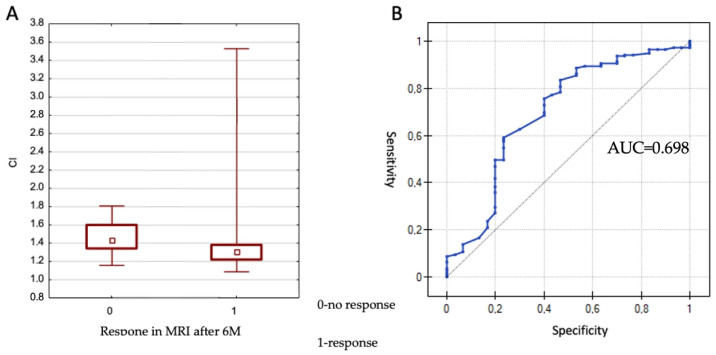
(**A**) Lesion response according to CI values. 0—no response confirmed on MRI, 1—response confirmed on MRI; (**B**) ROC curve for the CI parameter in assessing lesion response to treatment at 6 months. AUC calculated using DeLong’s method: 0.70 (95% CI: 0.58–0.81), *p* = 0.00069; optimal cut-off value determined using Youden’s index: CI = 1.42.

The CI (Conformity Index) values ranged from 1.09 to 2.97. The effect of CI on lesion control at 6 months was evaluated using the Mann–Whitney U test. No significant association was observed between CI and local control (*p* = 0.697). However, a statistically significant association was found between CI and treatment response (partial or complete response only) at 6 months (*p* = 0.001), as illustrated in Figure 1.

Given the statistical significance, a receiver operating characteristic (ROC) curve was constructed to determine the optimal CI cut-off value differentiating between response and non-response. The area under the curve (AUC) was 0.698, which differed significantly from 0.5 (*p* = 0.00069). The proposed cut-off value was 1.42, corresponding to a sensitivity of 83.5% and a specificity of 53.3%. In summary, better treatment responses were observed in lesions with a CI < 1.42.

### 3.4. Gradient Index (GI)

The GI (Gradient Index) values ranged from 2.43 to 5.78. The influence of GI on lesion control at 6 months was analyzed using the independent samples *t*-test. No statistically significant association was found (*p* = 0.599).

### 3.5. Distance to Isocenter (DTI)

**Figure 2:**
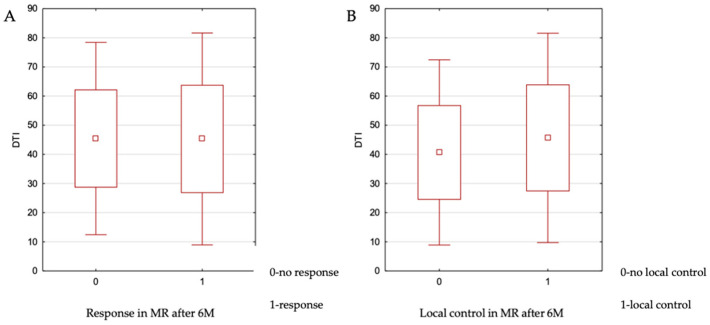
(**A**) No association was observed between DTI values and treatment response on MRI at 6 months after therapy. (**B**) No association was found between DTI values and local control on MRI at 6 months after treatment.

The DTI parameter for the analyzed metastatic lesions ranged from 7 to 85 mm, with a median value of 45 mm. The impact of DTI on local control at 6 months was assessed using the independent samples *t*-test. No statistically significant association was found (*p* = 0.967). The relationship between these variables is illustrated in Figure 2. Additionally, no significant relationship was observed between DTI and achieving a complete response versus partial response (*p* = 0.704) or complete response versus stable disease (*p* = 0.419).

### 3.6. Margins

**Figure 3:**
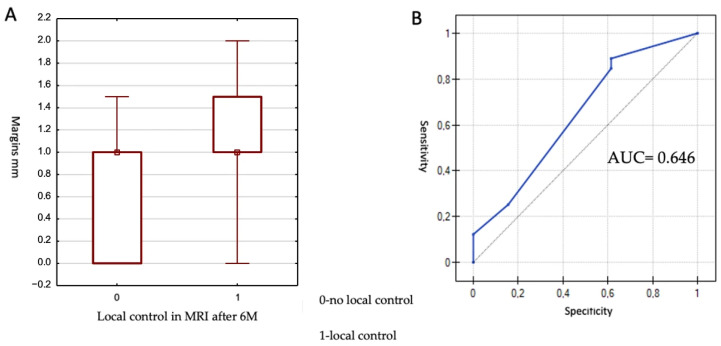
(**A**) Lesion local control according to CI values. 0—no local control confirmed on MRI, 1—response confirmed on MRI; (**B**) ROC curve for the margin parameter in assessing lesion local control to treatment at 6 months. AUC calculated using DeLong’s method: 0.646 (95% CI: 0.49–0.80), *p* = 0.079; optimal cut-off value determined using Youden’s index: cut-off = 0.5.

The smallest margin was 0 mm, and the largest was 2 mm, with a median of 1 mm. The Mann–Whitney U test was used for statistical analysis. A statistically significant impact of margin size on lesion control at 6 months was demonstrated (*p* = 0.049). The results are presented in Figure 3.

Due to the achievement of statistical significance, an attempt was made to determine a cut-off value for margin size associated with local control at 6 months. To this end, a receiver operating characteristic (ROC) curve was generated. The area under the ROC curve (AUC) was 0.646 and did not differ significantly from 0.5 (*p* = 0.079), indicating a borderline statistical significance. The proposed cut-off value was 0.5 mm, yielding a sensitivity of 89.0% and a specificity of 38.5%. In summary, lesions with a margin of at least 0.5 mm demonstrated better local control at 6 months post-treatment.

### 3.7. Dose

Doses ranging from 15 to 24 Gy in a single fraction were applied to the lesions. The specific dose used depended on the size and location of the tumor. The median dose administered was 20 Gy. The effect of the applied dose on lesion control at 6 months was analyzed. No statistically significant difference was observed (*p* = 0.360).

### 3.8. Effect of Immunotherapy or Targeted Therapy

**Figure 4:**
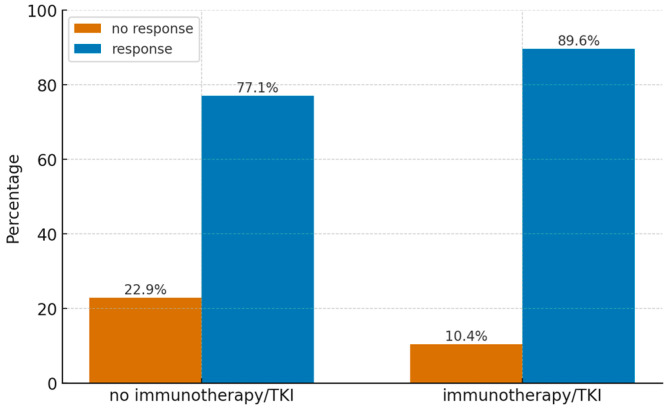
Distribution of response (blue) and no response (orange) in immunotherapy/TKI and non-immunotherapy/TKI groups.

In 13 patients (36%), immunotherapy or targeted therapy was administered within the first 4 months following stereotactic radiosurgery (SRS) for central nervous system (CNS) metastatic lesions. In all cases, immunotherapy or targeted therapy was administered sequentially, with systemic treatment initiated more than 2 weeks after SRS. The impact of systemic treatment on local control (defined as stabilization, partial response, or complete response) of the treated lesions was assessed. While no statistically significant association was confirmed, the result approached significance (*p* = 0.066). A similar analysis was performed for lesion response at 6 months (partial or complete response only), which revealed a statistically significant difference (*p* = 0.026). Data on the distribution of treatment response in patients with and without immunotherapy are presented in Figure 4.

An asymptotic odds ratio was calculated, yielding a statistically significant value of *p* = 0.030 for an odds ratio of 2.55 (95%CI 1.10; 5.98) indicating that patients who received immunotherapy or targeted therapy had a 2.55-fold greater likelihood of achieving a response compared to those who did not.

The influence of histopathological diagnosis on this relationship was also examined, with a statistically significant result observed (*p* = 0.0001). Based on prior analyses, a statistically significant association was observed (*p* < 0.001), with lung adenocarcinoma demonstrating better local control compared to other histopathological categories.

## 4. Discussion

The rising incidence of brain metastases and the increasing number of lesions per patient underscore the need for efficient and accurate treatment strategies. The DCA-SIMT approach enables simultaneous irradiation of multiple targets within a short treatment time but raises concerns regarding geometric accuracy, particularly for lesions located far from the isocenter. In a retrospective study, Kraft et al. [14] found that distance-to-isocenter (DTI) was not associated with local control, with one-year control rates exceeding 90% regardless of technique, while Aoki et al. [22] similarly reported no significant relationship between DTI and local failure in patients treated with single-isocenter, multi-target radiosurgery. Consistent with these findings, our results showed no correlation between DTI and either local control or radiographic response in the present cohort; however, the safety of treating lesions with very high DTI values remains uncertain and warrants further clinical validation.

The conformity index (CI) emerged as a more relevant predictor. Similarly to the reports of Aiyama and Yamamoto [23], we observed that lower CI values were associated with better local control. This is not surprising, as in clinical practice we strive to achieve lower CI values. However, when treating multiple small lesions with SIMT, high CI values are often encountered and are not optimal. An important practical implication of our analysis is the ability to define, within a large and representative cohort of lesions, a threshold CI value below which local control begins to decline. This finding may support treatment planning and plan acceptance processes, as it provides the opportunity to aim for a CI below the identified threshold in order to maintain therapeutic effectiveness. In contrast, the gradient index (GI) showed no association with local outcomes, consistent with previous studies.

Another modifiable factor is the margin expansion from GTV to PTV. While some centers omit margins altogether, particularly with Gamma Knife radiosurgery, linear accelerator-based approaches often incorporate 1–2 mm to account for intrafractional motion and image uncertainty. In our series, a margin of ≥0.5 mm appeared necessary to maintain local control, whereas larger margins (up to 2 mm) did not further improve outcomes and were not associated with an increased risk of radionecrosis. Kirkpatrick et al. [24], however, evaluated margins beyond 3 mm and reported a clear correlation with higher radionecrosis rates. These findings suggest that a minimal but non-zero margin may offer the optimal balance between efficacy and safety. Importantly, this information may be particularly valuable for treatment-planning decisions, allowing clinicians to select a minimal margin while maintaining adequate tumor coverage and treatment effectiveness. This issue is particularly relevant in the context of DTI, as the absence of a margin may be associated with poorer local control. Further studies are needed to determine whether the margin should be a function of DTI, since such an approach is increasingly used in clinical practice. Our results, however, do not provide evidence that margins larger than 0.5 mm are required. Some planning systems offer automatic margin selection based on DTI, but it remains to be investigated whether escalation is justified or whether a range of 0–0.5 mm in relation to DTI is sufficient.

Dose prescription remains a cornerstone of brain metastasis radiosurgery. Several studies have consistently reported improved outcomes with doses ≥ 18–24 Gy [25,26,27,28]. In our analysis, however, dose did not significantly affect local control. This likely reflects the relatively narrow dose range applied (15–24 Gy, median 20 Gy) and the small number of lesions treated with <16 Gy, which may have limited the ability to detect a dose–response relationship. The lack of an observed dose–effect relationship may also be related to the timing of assessment, as local failures typically occur later than six months after treatment. Given the short survival in patients with multiple metastases, however, evaluation at this time point remains clinically relevant.

The integration of stereotactic radiosurgery (SRS) with systemic therapy, particularly immunotherapy and targeted agents, is of increasing clinical relevance. In our cohort, systemic treatment administered within four months of SRS was associated with improved radiographic response, consistent with previous reports [29,30]. This effect may reflect enhanced drug delivery due to blood–brain barrier disruption during radiosurgery. While the optimal sequencing of SRS and systemic therapy remains uncertain, current data favor concurrent or post-SRS administration. Importantly, no excess toxicity was observed in patients receiving immunotherapy within four months after SRS. Future treatment strategies and clinical research should be based on the synergistic interaction between SRS and systemic therapy. Further studies are needed to determine the optimal timing of drug administration relative to SRS, potential dose–response relationships, and how these factors influence both local and systemic disease control.

This study has several limitations that should be acknowledged. First, its retrospective design and lack of randomization may introduce selection bias. Second, the relatively short follow-up period may limit the detection of late local failures. Additionally, the results may be influenced by confounding factors such as histopathological subtype and the availability of modern systemic therapies. Finally, the cohort size did not allow a separate evaluation of individual immunotherapy and targeted therapy agents, which should be further investigated in larger future studies.

## 5. Conclusions

Our findings confirm the efficacy of SIMT-based stereotactic radiosurgery for patients with multiple brain metastases, while underscoring the importance of conformity, minimal but sufficient margin expansion, and integration with systemic therapy as key determinants of outcome. Further studies with longer follow up and larger lesion cohort may provide us further insights on DTI and margin correlation as well as concomitant immunotherapy and SRS details.

## Figures and Tables

**Table 1:** Characteristics of treated patients and metastases.

Characteristic	Value
Number of patients	37
Age, years (median, IQR)	59 (45–77)
Sex (n, %)	
male	17 (46%)
female	20 (54%)
Number of metastases (median, IQR)	3 (2–11)
Mean GTV volume (cm^3^)	0.96
Mean PTV volume (cm^3^)	2.14
Primary tumor site (%)	
NSCLC	65%
breast	19%
melanoma	8%
other	8%
Dose, Gy (median, IQR)	20 (14–24)
Margins, mm (median, IQR)	1 (0–2)
Conformity Index (IQR)	1.09–2.97
Gradient Index (IQR)	2.43–5.78

**Table 2:** Treatment response of metastatic lesions after SRS; RICE—Radiation-Induced Contrast Enhancements.

Treatment Response	Number of Metastases
Stable (SD)	22 (11%)
Partial response (PR)	110 (56%)
Complete response (CR)	50 (26%)
Progression (PD)	1 (<1%)
RICE	12 (6%)

## Data Availability

The data supporting the findings of this study are available on reasonable request from the corresponding author.
